# Temporal Trends of Antithrombotic Therapy in Patients With Acute Myocardial Infarction and Atrial Fibrillation: Insight From the KAMIR-NIH Registry

**DOI:** 10.3389/fcvm.2021.762090

**Published:** 2021-11-25

**Authors:** Oh-Hyun Lee, Yongcheol Kim, Deok-Kyu Cho, Jung-Sun Kim, Byeong-Keuk Kim, Donghoon Choi, Myeong-Ki Hong, Myung Ho Jeong, Yangsoo Jang, the KAMIR-NIH Investigators

**Affiliations:** ^1^Division of Cardiology, Department of Internal Medicine, Yonsei University College of Medicine and Cardiovascular Center, Yongin Severance Hospital, Yongin, South Korea; ^2^Division of Cardiology, Severance Cardiovascular Hospital, Yonsei University College of Medicine, Seoul, South Korea; ^3^Division of Cardiology, Department of Internal Medicine, Chonnam National University Hospital, Gwangju, South Korea; ^4^Department of Cardiology, CHA Bundang Medical Center, CHA University, Seongnam, South Korea

**Keywords:** atrial fibrillation, myocardial infarction, percutaneous coronary intervention, anticoagulants, treatment outcome

## Abstract

**Background:** Triple therapy is the combination of dual antiplatelet therapy plus oral anticoagulant after stent implantation. Current guidelines recommend triple therapy for acute coronary syndrome with atrial fibrillation (AF). This study aimed to identify temporal trends of antithrombotic therapy in patients with acute myocardial infarction (AMI) and AF.

**Methods:** Among 13,104 consecutive patients from the Korea Acute Myocardial Infarction Registry-National Institute of Health (KAMIR-NIH) registry, we identified 453 patients with AF after stent implantation for AMI; these patients were then divided into those who did and did not use oral anticoagulant (OAC) [OAC group (*n* = 71) vs. non-OAC group (*n* = 382), respectively].

**Results:** The results showed that the prevalence of AF in AMI patients was 5.4% (712/13,104). Among 453 patients, only 15.7% (71/453) were treated with OAC while dual or single antiplatelet therapy was provided for 84.7% (382/453) of patients. In patients with high stroke risk (CHA_2_DS_2_-VASc score ≥ 2), OACs were used only in 17% (69/406). Multivariate analysis revealed that female sex [odds ratio (OR) 2.11; 95% CI: 1.17–3.79], diabetes mellitus (DM) (OR 2.37; 95% CI: 1.35–4.17), prior cerebrovascular accident (CVA) (OR 4.19; 95% CI: 2–8.75), and congestive heart failure (CHF) (OR 1.89; 95% CI: 1.09–3.3) as the significant determinants of OAC use.

**Conclusion:** The study concluded that OAC was underused. Approximately, 15%, of AMI patients with AF undergoing PCI with stent and female gender, DM, prior CVA history, and a history of CHF or the presence of moderate to severe left ventricle systolic impairment were significant determinants of OAC use.

## Introduction

Atrial fibrillation is the most common arrhythmia and is associated with increased morbidity and mortality, including thromboembolic events (1). The prevalence of atrial fibrillation (AF) is increasing globally and has been projected to increase to 5.6 million individuals in the USA by the year 2050 and 8.8 million adults over 55 years in Europe by the year 2060, indicating substantial public health and economic burden (2). AF is associated with an increased risk of acute myocardial infarction (AMI) and the presence of AF during AMI has been associated with a worse prognosis (3, 4).

In AF patients, oral anticoagulant (OAC) is required to reduce the risk of thromboembolic events, whereas antiplatelet therapy is essential to prevent thrombotic events, including stent thrombosis, in patients with ischemic heart disease undergoing percutaneous coronary intervention (PCI). However, there are concerns regarding the major bleeding due to the intensive combination antithrombotic therapy (5, 6). Thus, current guidelines recommend that early cessation of aspirin and continuation of dual antithrombotic therapy with an OAC plus clopidogrel up to 6–12 months in patients with AF at increased risk of stroke (CHA_2_DS_2_-VASc score ≥ 2) who have undergone PCI with stent implantation for ischemic heart disease (7, 8).

However, despite the recommendations of the current guidelines, a previous nationwide population-based study reported that only 22.7% of patients received triple therapy, i.e., the combination of dual antiplatelet therapy (DAPT) plus OAC, after PCI although 96.2% of them were indicated for anticoagulation (9). Until now, there is a paucity of data regarding the status of anticoagulant usage in AF patients who underwent PCI with stent implantation for AMI. Therefore, this study aimed to identify the trends in OAC usage patterns and the variables associated with the use of OAC from the real world in patients with AF presenting AMI who underwent PCI with stent implantation.

## Materials and Methods

### Study Design and Subjects

The study population in the current study was selected from the Korea Acute Myocardial Infarction Registry-National Institute of Health (KAMIR-NIH) registry, a nationwide prospective multicenter registry of patients with AMI in the Republic of Korea without any exclusion criteria. Furthermore, 20 tertiary university hospitals with facilities for primary PCI and onsite cardiac surgery participated in this registry. The detailed study protocol has been previously published (10). After discharge, clinical follow-up was carried out by patient visits or telephone interviews at 6 and 12 months. All data were assessed by independent clinical research coordinators using a web-based case report form on the internet-based Clinic Research and Trial management system. Clinical outcomes were monitored and centrally adjudicated by an independent event adjudication committee.

Among the consecutive patients with AMI enrolled from November 2011 to December 2015, we excluded the patients who died during the hospitalization for AMI and patients who were lost to follow-up during the 1-year follow-up period.

This study complied with the provisions of the Declaration of Helsinki. The study protocol was approved by the Institutional Review Board of each participating center. The approval number was CNUH-2011-172 at Chonnam National University Hospital. All patients provided written informed consent.

### Definitions

Congestive heart failure (CHF)/left ventricular dysfunction, hypertension, age ≥75 years (doubled), diabetes, stroke (doubled)-vascular disease, 65–74 years of age, and sex category (female) [CHA_2_DS_2_-VASc]. Score was calculated for all patients (8, 11). In this score, the C was defined as the history of congestive heart failure (CHF) or the presence of moderate to severe LV systolic impairment [left ventricular ejection fraction (LVEF) ≤ 40%] on transthoracic echocardiography. According to a major criterion of the Academic Research Consortium for High Bleeding Risk (ARC-HBR), anemia and thrombocytopenia were defined as the hemoglobin level <11 g/dl and platelet count <100 × 10^9^/L, respectively (12). Severe chronic kidney disease was defined as a severe or end-stage renal disease, with an estimated glomerular filtration rate (eGFR) <30 ml/min (12).

### Statistical Analysis

Normally distributed continuous variables are expressed as *M* ± *SD*. All categorical variables were presented as numbers with percentile values. Time-to-event data are presented using Kaplan-Meier estimates. The hazard ratios (HR) with 95% CI were estimated from a Cox proportional hazards model. To identify the determinants for the use of OAC, we performed univariate and multivariate analyses. Any variable with a *p* < 0.10 on univariate analysis was included in the multivariate models. Comparison of clinical outcomes between two groups was assessed after adjustment for multiple risk factors using the logistic regression model with the inverse probability of treatment weighting (IPTW). All statistical analyses were performed with SPSS statistical software (SPSS version 25 for Windows; IBM Corp., Armonk, NY, USA).

## Results

**Figure 1:**
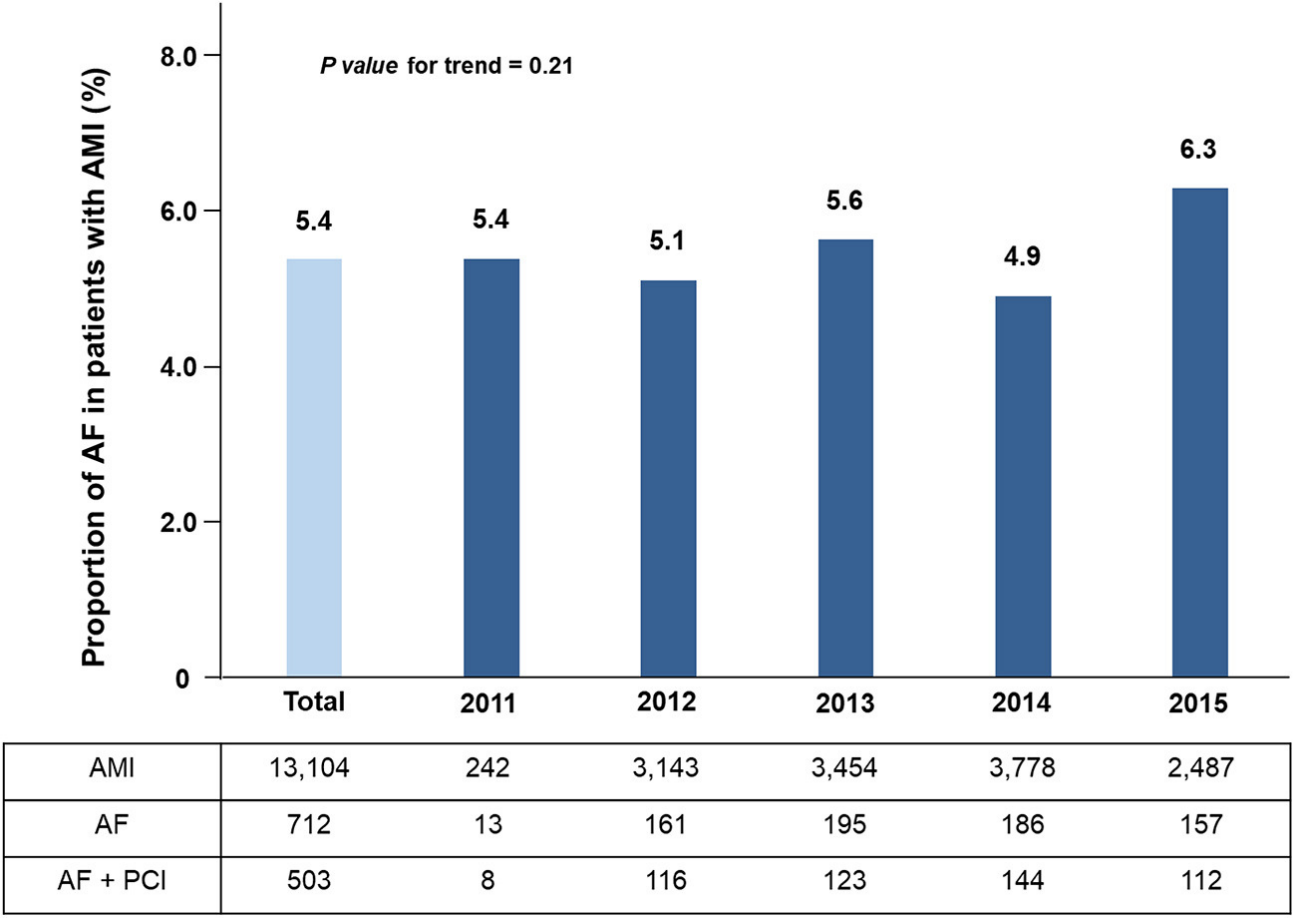
The trend of annual AF incidence in patients with acute myocardial infarction.

Among the entire 13,104 patients from the KAMIR-NIH registry, the prevalence of AF was 5.4% (712/13, 104) and the trend of annual AF incidence in patients with AMI was stable (*p-*value for trend = 0.21) (Figure 1). Of these patients, 17% (121/712) patients have been newly diagnosed AF after AMI. A total of 453 eligible patients with AF and AMI treated with stent implantation were analyzed. The mean age was 68.5 ± 12 years (range: 37–92 years), and 337 (74.4%) of the patients were men. The mean CHA_2_DS_2_-VASc Score was 3.7 ± 1.7.

Among 453 patients who underwent PCI with stent implantation successfully and were discharged, only 15.7% of patients were treated with OAC although 89.6% of them were indicated for anticoagulation (CHA_2_DS_2_-VASc score ≥ 2). The study population was classified into two groups according to the OAC usage after stent implantation as follows: patients who used OAC according to the clinical guidelines (*n* = 71, 15.7%) and did not use OAC (*n* = 382, 84.3%) (Supplementary Figure 1). Baseline characteristics of the study population are presented in Table 1. Compared with patients treated without OAC, those using OAC were higher rates of female, diabetes mellitus, current smoker, a history of cerebrovascular accident, and moderate to severe left ventricular systolic dysfunction. Aspirin and clopidogrel were used more frequently in OAC groups, while ticagrelor was used more often in the non-OAC group than in the OAC group. Other baseline characteristics were comparable between the two groups.

**Table 1:** Baseline characteristics.

**Characteristics**	**OAC** **(***n*** = 71)**	**No OAC** **(***n*** = 382)**	***p*** **-value**
Age, years	70.6 ± 10.5	68.1 ± 12.3	0.08
Female gender	27 (38.0)	89 (23.3)	0.01
Height, cm	163.2 ± 9.1	165.2 ± 8.6	0.08
Weight, kg	65.2 ± 10.9	65.3 ± 11.78	0.95
Body mass index, kg/m^2^	24.3 ± 2.0	23.8 ± 3.5	0.24
Hypertension	49 (69.0)	217 (56.8)	0.06
Diabetes mellitus	33 (46.5)	88 (23.0)	<0.01
Dyslipidemia	9(12.7)	26 (6.8)	0.09
Current smoking	11 (15.5)	133 (34.8)	<0.01
Prior myocardial infarction	6 (8.5)	27 (7.1)	0.68
Prior cerebrovascular accident	17 (23.9)	23 (6.0)	<0.01
Prior congestive heart failure	5 (7.0)	12 (3.1)	0.16
**Clinical presentation**			
NSTEMI	38 (53.5)	163 (42.7)	0.09
STEMI	33 (46.5)	219 (57.3)	
Killip class 3/4	17 (23.9)	86 (22.5)	0.79
LVEF, %	47.3 ± 11.7	51.0 ± 10.7	0.01
LVEF ≤ 40%	22 (31.0)	53 (13.9)	<0.01
**Vital sign**			
Systolic BP, mmHg	122.5 ± 29.6	119.3 ± 34.1	0.46
Diastolic BP, mmHg	76.1 ± 20.1	72.8 ± 21.2	0.23
Heart rate, beats per min	88.6 ± 20.6	81.3 ± 27.2	0.01
**Laboratory findings**			
Peak troponin I, pg/ml	47.0 ± 76.4	51.0 ± 84.6	0.73
Total cholesterol, mg/dL	151.5 ± 48.5	168.2 ± 39.0	0.01
Triglyceride, mg/dL	104.0 ± 64.8	117.2 ± 81.5	0.22
HDL-cholesterol, mg/dL	41.4 ± 13.7	42.9 ± 12.0	0.39
LDL-cholesterol, mg/dL	95.1 ± 38.3	103.7 ± 35.5	0.08
Creatinine, mg/dL	1.2 ± 1.5	1.2 ± 0.9	0.91
Hemoglobin, g/dL	13.5 ± 2.1	13.8 ± 2.1	0.35
Platelet count, 10^3^/μL	219.3 ± 63.6	213.7 ± 60.0	0.47
CRP, mg/L	1.7 ± 4.3	1.9 ± 7.6	0.85
NT-proBNP, pg/mL	4195.7 ± 5662.6	4007.1 ± 12351.7	0.91
**Discharge medication**			
Aspirin	68 (95.8)	381 (99.7)	0.01
P2Y_12_ inhibitor	70 (98.6)	378 (99.0)	0.58
Clopidogrel	64 (90.1)	288 (75.4)	<0.01
Prasugrel	3 (4.2)	30 (7.9)	0.45
Ticagrelor	3 (4.2)	60 (15.7)	<0.01
ACEi or ARB	59 (83.1)	299 (78.3)	0.36
Beta-blocker	55 (77.5)	315 (82.5)	0.32
Calcium channel blocker	5(14.7)	29 (7.6)	1.00
Statin	63 (88.7)	360 (94.2)	0.09

*Data are presented as the Mean ± SD or number (%)*.

*OAC, oral anticoagulant; NSTEMI, non-ST-segment elevation myocardial infarction; STEMI, ST-segment elevation myocardial infarction; LVEF, left ventricular ejection fraction; BP, blood pressure; HDL, high-density lipoprotein; LDL, low-density lipoprotein; CRP, C-reactive protein; NT-proBNP, N-terminal pro-B-type Natriuretic peptide; ACEi, angiotensin-converting enzyme inhibitor; ARB, angiotensin receptor blocker*.

Angiographic and procedural characteristics are listed in Supplementary Table 1. Among these, disease extent, the total number of implanted stents, mean stent diameter, and total stent length did not significantly differ between the two groups, except for the distribution of infarct-related arteries.

**Figure 2:**
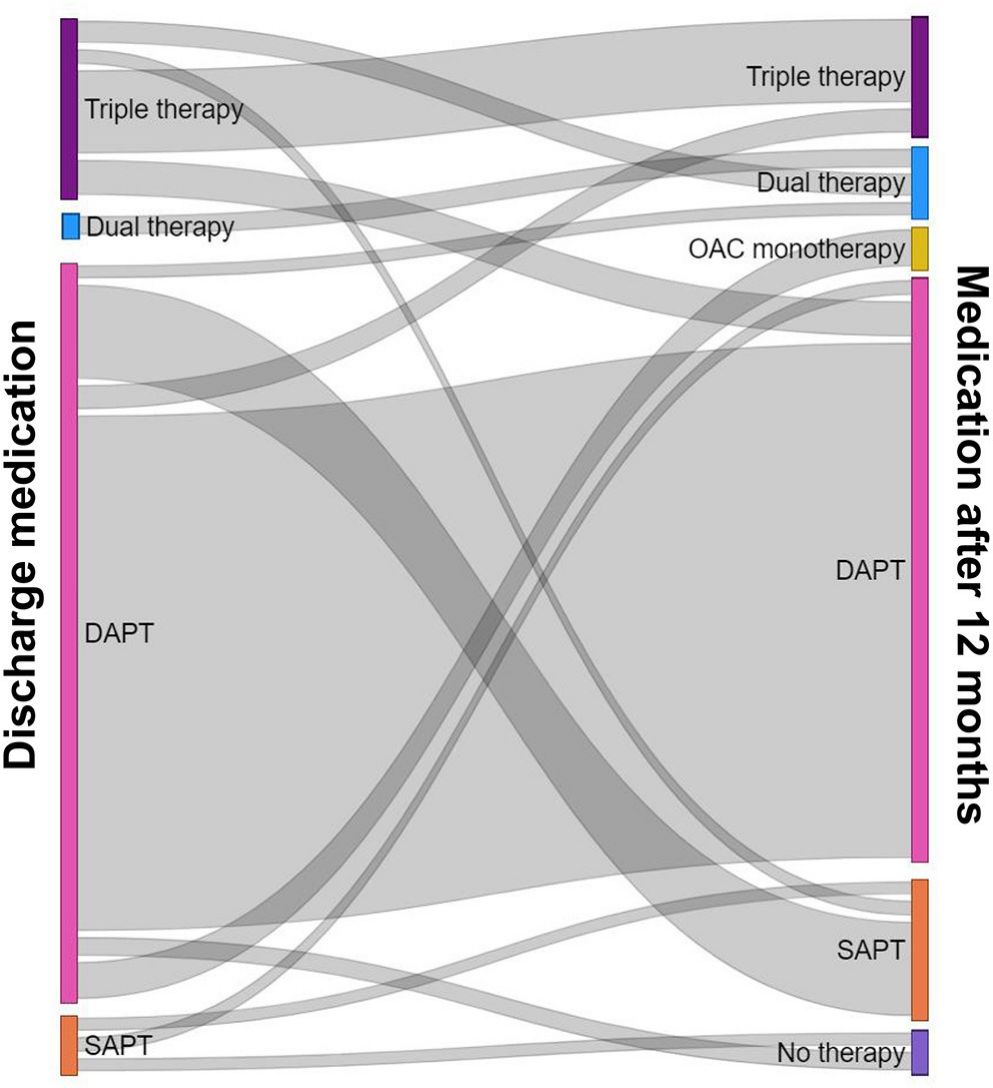
Detailed information on the antithrombotic treatment at discharge and 12 months after PCI for AMI. PCI, percutaneous coronary intervention; AMI, acute myocardial infarction.

Regarding the trends of antithrombotic therapy in both groups, among the 71 patients in the OAC group, triple therapy was used in 67 (94.4%) patients. The majority of patients with triple therapy received clopidogrel (61/67). Only four patients in the OAC group were treated with dual therapy. In patients treated without OAC, 378 (99%) patients received DAPT and four (1%) patients treated with single antiplatelet treatment (SAPT) (Table 2). Detailed information on the antithrombotic treatment at discharge and after 12 months is summarized in Table 3 and Figure 2.

**Table 2:** Detailed information on antithrombotic therapy.

**Characteristics**	**OAC** **(***n*** = 71)**		**No OAC** **(***n*** = 382)**
Triple therapy	67 (94.4)	Dual antiplatelet therapy	378 (99.0)
OAC + Aspirin + Clopipdogrel	61 (85.9)	Aspirin + Clopipdogrel	288 (75.4)
OAC + Aspirin + Prasugrel	3 (4.2)	Aspirin + Prasugrel	30 (7.9)
OAC + Aspirin + Ticagrelor	3 (4.2)	Aspirin + Ticagrelor	60 (15.7)
OAC + single antiplatelet therapy	4 (5.6)	Single antiplatelet therapy	4 (1.0)
OAC + Aspirin	1 (1.4)	Aspirin	4 (1.0)
OAC + Clopidogrel	3 (4.2)	Clopidogrel	0
OAC monotherapy	0	No antithrombotic therapy	0

*Data are presented as the number (%). OAC, oral anticoagulant*.

**Table 3:** Changes of antithrombotic trends between discharge and 12-month follow-up visit.

**Medication at discharge**	***N*** **= 453**	**Medication at 12-month**	***N*** **= 453**
Triple therapy	67 (14.8)	Triple therapy	46 (10.2)
Dual therapy	4 (0.9)	Dual therapy	11 (2.4)
OAC monotherapy	0	OAC monotherapy	14 (3.1)
Dual antiplatelet therapy	378 (83.4)	Dual antiplatelet therapy	288 (63.6)
Single antiplatelet therapy	4 (0.9)	Single antiplatelet therapy	48 (10.6)
No antithrombotic therapy	0	No antithrombotic therapy	5 (1.1)
		No available data	41 (9.1)
**Detail changes of antithrombotic trends in patients treated with triple therapy at discharge**			
Triple therapy	67 (14.8)	Triple therapy	39 (58.2)
		Dual therapy	6 (9.0)
		OAC monotherapy	0
		Dual antiplatelet therapy	13 (19.4)
		Single antiplatelet therapy	2 (3.0)
		No antithrombotic therapy	0
		No available data	7 (10.4)

*Data are presented as the number (%)*.

**Figure 3:**
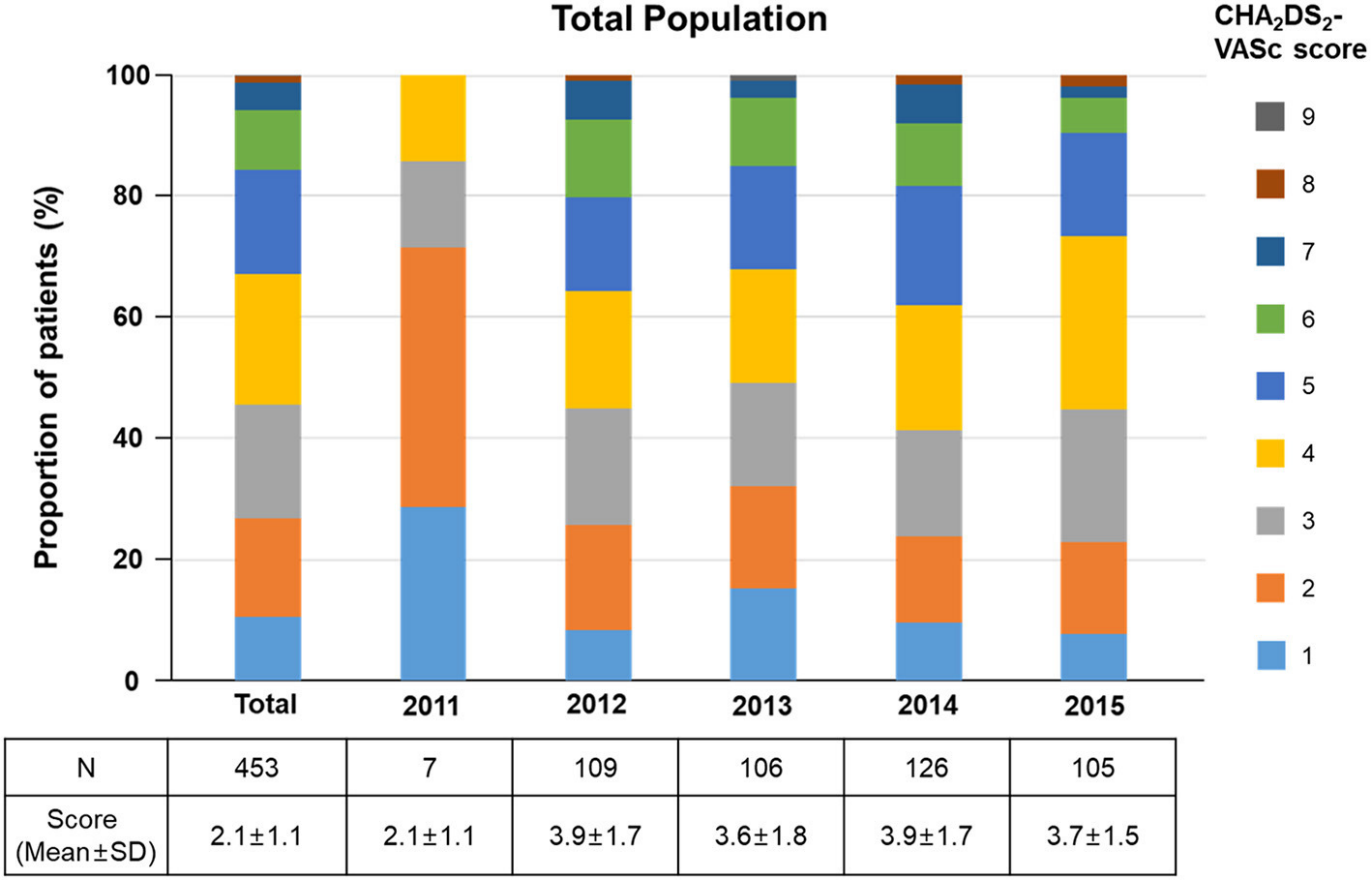
The trend of detailed distributions of the CHA2DS2-VASc scores.

The scores in CHA_2_DS_2_-VASc of the study population are described in Table 4. Mean CHA_2_DS_2_-VASc score was significantly higher in the OAC group than in the non-OAC group (4.7 ± 1.6 vs. 3.6 ± 1.7, *p* < 0.01), and the proportion of patients with a score of 2 or more were much higher in the OAC than in the non-OAC group [97.2% (69/71) vs. 88.2% (337/382), *p* = 0.02] (Supplementary Figure 2). The detailed distributions of the CHA_2_DS_2_-VASc scores are shown in Figure 3. Among the 453 study patients, 406 had a high risk of stroke (CHA_2_DS_2_-VASc Score ≥ 2). However, OAC was used only in 17% (69/406). In the low to intermediate stroke risk group (CHA_2_DS_2_-VASc Score <2), 4.3% (2/47) of patients were treated with OAC. Among the components of the CHA_2_DS_2_-VASc score, the OAC group had a higher prevalence of CHF, diabetes mellitus, prior stroke, TIA or thromboembolism history, age 65–74 years, and female gender (Table 4).

**Table 4:** Detail information of CHA_2_DS_2_-VASc Score.

**Characteristics**	**OAC** **(***n*** = 71)**	**No OAC** **(***n*** = 382)**	***p*** **value**
**Index**			
C CHF	40 (56.3)	148 (38.7)	<0.01
H Hypertension	49 (69.0)	217 (56.8)	0.055
A2 Age ≥ 75 years	25 (35.2)	136 (35.6)	0.95
D Diabetes mellitus	33 (46.5)	88 (23.0)	<0.01
S2 Prior stroke or TIA or thromboembolism	17 (23.9)	23 (6.0)	<0.01
V Vascular disease	71 (100)	382 (100)	–
A Age 65–74 years	32 (45.1)	113 (29.6)	0.01
Sc female gender	27 (38.0)	89 (23.3)	<0.01
CHA_2_DS_2_-VASc Score	4.7 ± 1.6	3.6 ± 1.7	<0.01
Score ≥ 2	69 (97.2)	337 (88.2)	0.02
Score ≥ 3	66 (93.0)	266 (69.6)	<0.01
Score ≥ 4	55 (77.5)	192 (50.3)	<0.01
Score ≥ 5	40 (56.3)	109 (28.5)	<0.01
Score ≥ 6	25 (35.2)	46 (12.0)	<0.01
Score ≥ 7	7 (9.9)	19 (5.0)	0.10

*Data are presented as the mean ± SD or number (%)*.

*OAC, oral anticoagulant; CHF, congestive heart failure; TIA, transient ischemic attack*.

In the multivariate analyses for the determinants of the use of OAC, female gender, diabetes mellitus, and prior CVA history were significant determining factors. History of CHF or the presence of moderate to severe left ventricle (LV) systolic impairment (LVEF ≤ 40%) was also a significant determinant (Table 5).

**Table 5:** Univariate and multivariate analyses regarding the determinants for OAC usage.

**Variables**	**Univariate analysis**		**Multivariate analysis^†^**	
	**OR (95% CI)**	***P*** **-value**	**OR (95% CI)**	***P*** **-value**
Age ≥ 75 years	0.98 (0.58–1.67)	0.95		
Body mass index, kg/m^2^	1.05 (0.97–1.13)	0.24		
Female gender	2.02 (1.18–3.45)	0.01	2.11 (1.17–3.79)	0.01
Hypertension	1.69 (0.99–2.91)	0.06	1.02 (0.56–1.87)	0.95
Diabetes mellitus	2.90 (1.72–4.90)	<0.01	2.37 (1.35–4.17)	<0.01
Dyslipidemia	1.99 (0.89–4.44)	0.09	1.77 (0.73–4.29)	0.21
Prior CVA	4.91 (2.47–9.79)	<0.01	4.19 (2.00–8.75)	<0.01
CKD ≥ stage 4 (eGFR <30)	1.03 (0.34–3.09)	0.96		
NSTEMI	1.55 (0.93–2.57)	0.09	1.46 (0.84–2.53)	0.18
CHA_2_DS_2_-VASc Score ≥ 2	4.61 (1.09–19.44)	0.04	1.51 (0.32–7.21)	0.60
CHF	2.04 (1.22–3.41)	<0.01	1.89 (1.09–3.30)	0.02
Anemia (Hb <11 g/dL)	0.98 (0.39–2.42)	0.96		
Thrombocytopenia (platelet count <100 × 10^9^/L)	0.77 (0.09–6.32)	0.80		
LM involvement	1.14 (0.38–3.46)	0.82		
Stent number	0.92 (0.52–1.65)	0.79		
Mean stent diameter	0.88 (0.49–1.57)	0.67		
Total stent length	0.99 (0.98–1.02)	0.84		

† *Any variable with P < 0.1 on univariate analysis was included in the multivariate models*.

*OAC, oral anticoagulant; CVA, cerebrovascular accident; eGFR, estimated glomerular filtration rate; CHF, congestive heart failure; Hb, hemoglobin; LM, left main artery*.

During a 1-year follow-up, there were 44 major adverse cardiovascular and cerebrovascular events (MACCE) (28 all-cause deaths, five MIs, and six cerebral infarctions or TIAs) after stent implantation, and no significant difference was noted between the OAC and non-OAC groups in both the crude- and IPTW-analyses (Supplementary Table 2). The rates of MACCE, composite all-cause death and MI, composite cardiac death, and MI were also not significantly different between the two groups in both the crude- and IPTW-analyses.

## Discussion

The principal findings of the current study were as follows: (1) in real-world practice, the prevalence of AF in AMI patients was 5.4%; (2) only 15.7% of total AMI patients were treated with OAC although 89.6% of them were indicated for anticoagulation (CHA_2_DS_2_-VASc score ≥ 2); (3) the significant determinants for the use of OAC were female gender, diabetes mellitus, prior CVA history, and a history of CHF or the presence of moderate to severe LV systolic impairment.

The prevalence of AF in the entire Korean population was reported to be 67% in a previous study using the National Health Insurance Service database from 2008 to 2015 and the incidence and prevalence of AF increased in the Korean population over time (13). However, the prevalence of AF in Korean AMI patients has not been reported to date. Previous studies with the western population reported that AF occurred in 7.2–13.2% of patients with AMI (14, 15). In the China registry, the prevalence of AF was reported to be 3% in AMI patients (16). In the current nationwide study, the prevalence of AF in AMI patients was 5.4% and the trend of annual AF incidence in patients with AMI was stable. A previous study showed that the prevalence of AF was significantly higher in Caucasian (8%) than in black (3.8%), Hispanic (3.6%), and Asian (3.9%) ethnic groups (17). Therefore, our study results supported that the prevalence of AF in Asian patients with AMI was relatively lower when compared with Caucasians although more data should be collected (16).

Although the presence of AF during AMI has been associated with a worse prognosis (4), OAC was underused, in only ~15% of the AMI patients with AF undergoing PCI with a stent in the current study. Previous US registry reported only 27% (*n* = 448) of the total population received triple therapy at discharge among the 1,648 patients with AF with non-ST-elevation myocardial infarction who underwent PCI (18). The Swedish registry also reported the only 30% (1,848/6,182) of total AMI patients with AF admitted from 1995 to 2002 were prescribed OAC (19). The tendency of underused OAC in the current and previous studies could be explained by the concern regarding bleeding complications when patients were treated with triple therapy. Previous nationwide cohort study with AF patients following AMI and PCI showed that triple therapy increased both early and delayed bleeding risk compared with DAPT (20). Therefore, the concern for major bleeding may have affected the decision of physicians to not use OAC. However, since the introduction of non-vitamin K oral anticoagulants (NOACs), recent studies consistently showed a significant reduction in major bleeding with NOAC-based treatment compared with the vitamin K antagonist-based therapies, resulting in a rapid increase in triple therapy with NOAC after PCI for AMI in AF patients (21). As a result, the current guidelines recommend the preferable use of NOAC over vitamin K antagonists (21). In the era of NOAC, further study regarding recent trends of antithrombotic therapy in patients with AF and AMI is needed.

Cardiac catheterization with radial access has been reported to be more beneficial than femoral access with fewer occurrences of hemorrhagic events and short-term cardiac death (22). In the current study, only 27.6% (125/453) of total patients underwent PCI *via* transradial access. The proportion of transfemoral access is somewhat high, which may have affected the operator to be reluctant to prescribe OAC.

There are concerns regarding the major bleeding due to the intensive combination antithrombotic therapy. Thus, current guidelines recommend that early cessation of aspirin and continuation of dual antithrombotic therapy with an OAC plus clopidogrel up to 6–12 months in patients with AF at increased risk of stroke (CHA_2_DS_2_-VASc score ≥ 2) who have undergone PCI with stent implantation for ischemic heart disease (7, 8). Although this study was a study of patients from November 2011 to December 2015, the ESC guideline for STEMI published in 2012 also strongly recommended triple therapy with minimal duration to reduce bleeding risk in patients with obligatory indication for OAC after stent placement (Class 1, Level C) (23). However, unlike the recommendation of the guidelines, 14.8% (67/453) of AF patients who underwent PCI for AMI were treated with triple therapy at discharge, of which the majority of patients (58.2%) maintained triple therapy for 1 year. Therefore, further research and discussion on this area are needed.

A previous nationwide population-based study reported that the prior CVA history and congestive heart failure were significantly associated with a higher triple prescription rate in patients with AF undergoing PCI (9). In the current study, we also found that the prior CVA history and a history of CHF or the presence of moderate to severe LV systolic impairment were significant determinants for physicians to the use of OAC. Additionally, female gender and diabetes mellitus were significant factors affecting OAC usage. Among the components of the CHA_2_DS_2_-VASc score, our study results showed that older age and hypertension did not affect the use of OAC. This might be explained by the fact that previous studies, including the current study, were for patients with ischemic heart disease and AF, rather than overall AF patients, indicating different characteristics of OAC usage.

This study had several limitations. First, this study was a non-randomized, observational study, which has inherent selection and information biases. Second, the current study was conducted on patients registered in the registry, thus the results were difficult to apply with representation and in hypothesis generation. Third, details of the prescription of the OAC such as treatment duration of OAC and type of OAC (vitamin K antagonist or NOAC) were not considered. However, we identified the trend of change in antithrombotic therapy between the hospital discharge and 12 months after PCI. Fourth, currently available data did include only in-hospital bleeding events. Fifth, the comparison of 12-month clinical outcome was done by classifying the patients based on OAC usage at the time of hospital discharge without considering the treatment duration or type of OAC, or concomitant antiplatelet agents, so the results should be interpreted with caution. Nevertheless, this study expects physicians to be able to understand the trends in the use of OAC usage in patients with AMI and AF.

## Conclusion

The nationwide prospective dedicated AMI registry demonstrated that the prevalence of AF was 5.4% in patients with AMI. Moreover, contrary to the guidelines, OAC was underused, in only ~15% of the AMI patients with AF undergoing PCI with a stent in real-world practice. The significant determinants for the use of OAC were female gender, diabetes mellitus, prior CVA history, and a history of CHF or the presence of moderate to severe LV dysfunction, although the older age and hypertension did not affect the use of OAC among the components of the CHA_2_DS_2_-VASc score.

## Data Availability Statement

The data supporting the conclusions of this article are available from the corresponding author upon reasonable request.

## Ethics Statement

The studies involving human participants were reviewed and approved by Institutinal Review Board of each participating center. The approval number was CNUH-2011-172 at Chonnam National University Hospital. The patients/participants provided their written informed consent to participate in this study.

## Author Contributions

O-HL and YK study concept and design, acquisition, analysis, and interpretation of data, drafting for the manuscript, and critical revision of the manuscript for important intellectual content. YK, D-KC, J-SK, B-KK, DC, M-KH, MJ, and YJ acquired the data. D-KC, DC, M-KH, and YJ supervised the progress of the study. All authors listed have made a substantial, direct and intellectual contribution to the work, and approved it for publication.

## Funding

This work was supported by a faculty research grant from Yonsei University College of Medicine (6-2020-0161) and a research seed money grant of Internal Medicine in Yongin Severance Hospital.

## Conflict of Interest

The authors declare that the research was conducted in the absence of any commercial or financial relationships that could be construed as a potential conflict of interest.

## Publisher's Note

All claims expressed in this article are solely those of the authors and do not necessarily represent those of their affiliated organizations, or those of the publisher, the editors and the reviewers. Any product that may be evaluated in this article, or claim that may be made by its manufacturer, is not guaranteed or endorsed by the publisher.
