# Supplementary material for: Temporal Trends of Antithrombotic Therapy in Patients With Acute Myocardial Infarction and Atrial Fibrillation: Insight From the KAMIR-NIH Registry
Source: Front Cardiovasc Med. 2021 Nov 25;8:762090. doi: 10.3389/fcvm.2021.762090 (PMC8655723; doi:10.3389/fcvm.2021.762090)
Supplement: Supplementary file 1 [file Data_Sheet_1.docx]

**SUPPLEMENTARY MATERIALS**

**Temporal trends of antithrombotic therapy in patients with acute myocardial infarction and atrial fibrillation: insight from the KAMIR-NIH registry**

**Supplement to: OH Lee, Y Kim, DK Cho, et al., on behalf of the KAMIR-NIH Investigators**

**CONTENTS**

**SUPPLEMENMTARY FIGURE S1. Study flow chart** ...………………..……….………..2

**SUPPLEMENMTARY FIGURE S2. Comparison in proportion of patients of CHA_2_DS_2_-VASc score ≥ 2**..……………………………………………………………………………….3

**SUPPLEMENMTARY TABLE S1. Angiographic and procedural characteristics**….......4

**SUPPLEMENMTARY TABLE S2. One-year clinical outcomes between OAC and non-OAC groups.** .............................................................................................................................5

**Supplementary Figure S1. Study flowchart.**

**
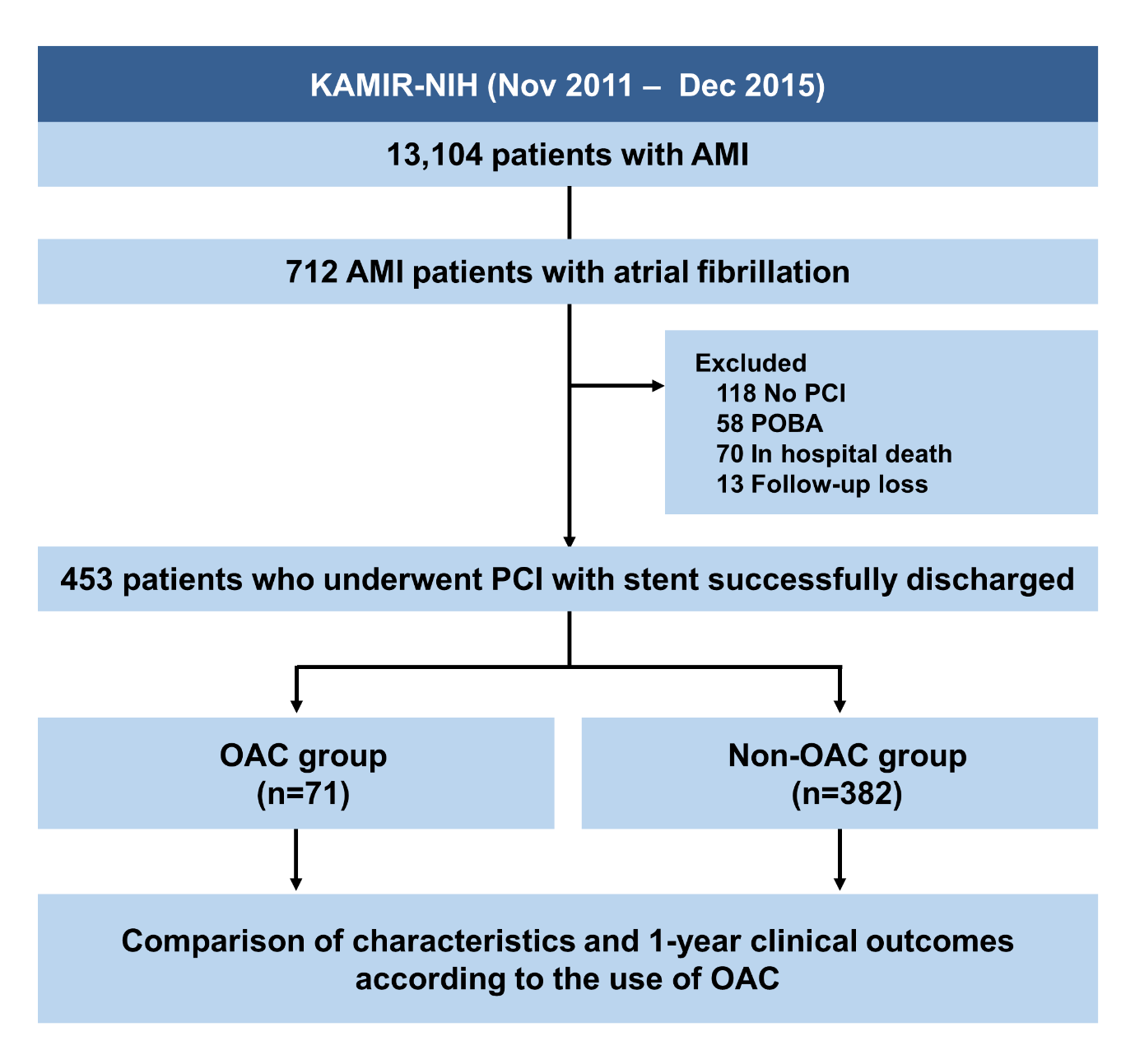
**

**Supplementary Figure S2.** Comparison in proportion of patients of CHA_2_DS_2_-VASc score ≥ 2.

**
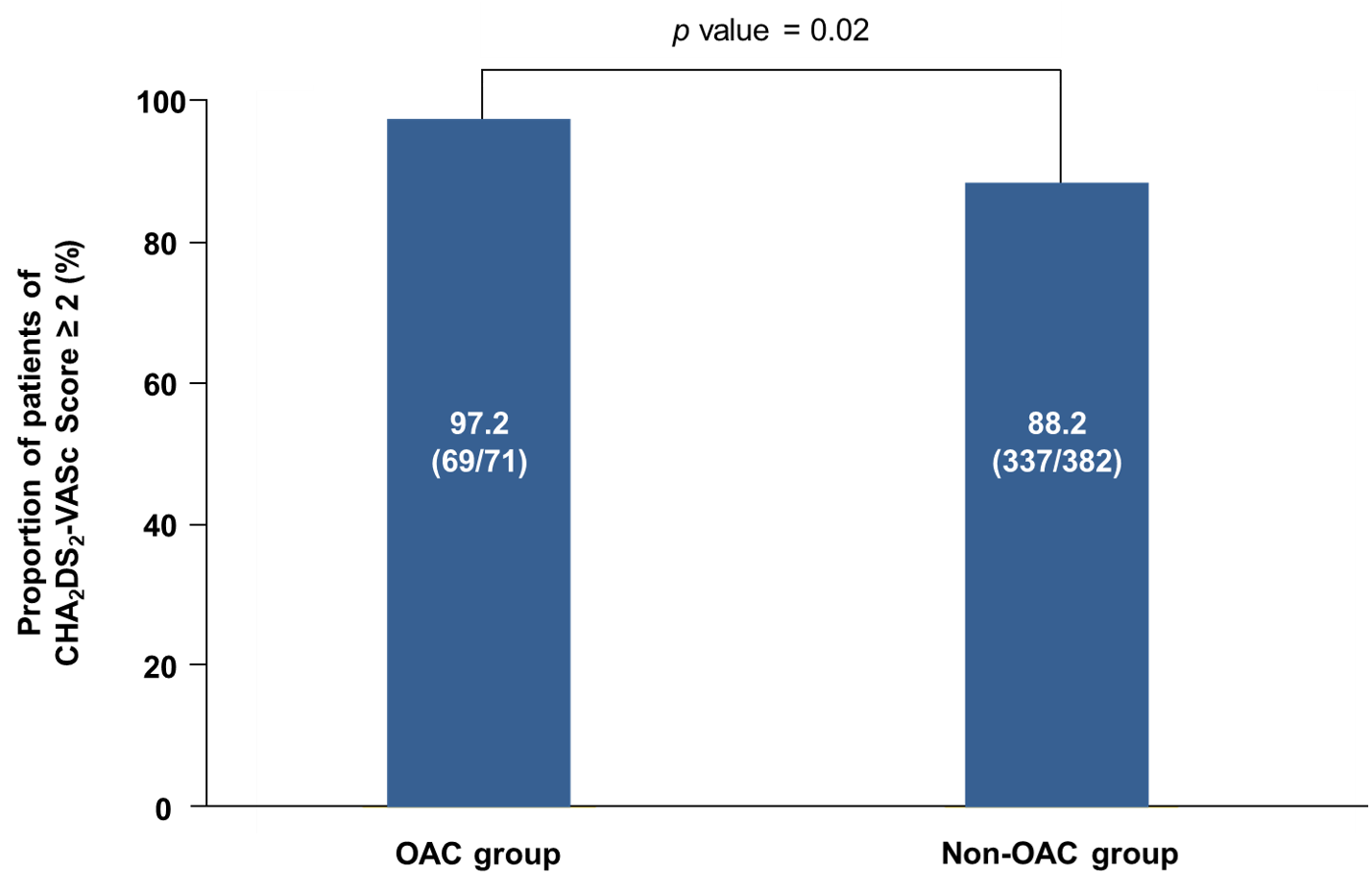
**

**Supplementary Table S1. Angiographic and procedural characteristics**

| Characteristics | OAC (n=71) | No OAC (n=382) | *p* value |
| --- | --- | --- | --- |
| Trans-radial approach | 25 (35.2) | 100 (26.2) | 0.12 |
| Target vessel |  |  |  |
| LM | 3 (4.2) | 10 (2.6) | 0.02 |
| LAD | 38 (53.5) | 136 (35.6) |  |
| LCX | 7 (9.9) | 72 (18.8) |  |
| RCA | 23 (32.4) | 164 (42.9) |  |
| CAD extent |  |  |  |
| CAD 1VD | 38 (53.5) | 177 (46.3) | 0.52 |
| CAD 2VD | 22 (31.0) | 131 (34.3) |  |
| CAD 3VD | 11 (15.5) | 74 (19.4) |  |
| LM involvement | 4(5.6) | 19 (5.0) | 0.77 |
| B2/C lesion | 65 (91.5) | 343 (89.8) | 0.65 |
| Stent number | 1.2±0.4 | 1.2±0.5 | 0.78 |
| Mean stent diameter | 3.12±0.44 | 3.14±0.45 | 0.67 |
| Total stent length | 28.9±12.9 | 29.3±13.7 | 0.84 |
| Thrombus aspiration | 11 (15.5) | 92 (24.1) | 0.11 |
| Glycoprotein IIb/IIIa inhibitor use | 9(12.7) | 78 (20.4) | 0.13 |

Data are presented as the mean ± SD or number (%)

Abbreviations: OAC, oral anticoagulant; LM, left main artery, LAD, left anterior descending artery; LCX, left circumflex artery; RCA, right coronary artery; CAD, coronary artery disease.

**Supplementary Table S2. One-year clinical outcomes between OAC and non-OAC groups.**

|  | Crude analysis | | | | IPTW analysis^†^ | | | |
| --- | --- | --- | --- | --- | --- | --- | --- | --- |
|  | OAC (n=71) | No OAC (n=382) | HR (95% CI) | *p* value | OAC (n=122) | No OAC (n=397) | HR (95% CI) | *p* value |
| MACCE (all-cause death, MI, and stroke) | 9 (7.4) | 35 (9.1) | 0.83 (0.40-1.72) | 0.62 | 9 (7.4) | 35 (9.1) | 0.83 (0.40-1.72) | 0.62 |
| Composite of all-cause death and MI | 9 (7.4) | 30 (7.8) | 0.97 (0.46-2.04) | 0.93 | 9 (7.4) | 30 (7.8) | 0.97 (0.46-2.04) | 0.93 |
| Composite of cardiac death and MI | 8 (6.6) | 24 (6.4) | 1.08 (0.48-2.39) | 0.86 | 8 (6.6) | 24 (6.4) | 1.08 (0.48-2.39) | 0.86 |
| All-cause mortality | 5 (7.0) | 23 (6.2) | 1.15 (0.44-3.03) | 0.77 | 9 (7.4) | 27 (7.0) | 1.07 (0.51-2.28) | 0.85 |
| Cardiac death | 4 (5.7) | 18 (4.9) | 1.19 (0.40-3.50) | 0.76 | 8 (6.6) | 21 (5.5) | 1.23 (0.55-2.79) | 0.61 |
| Noncardiac death | 1 (1.5) | 5 (1.3) | 1.06 (0.12-9.09) | 0.96 | 1 (0.9) | 6 (1.6) | 0.54 (0.07-4.47) | 0.57 |
| Cerebral infarction/TIA | 1 (1.4) | 5 (1.4) | 1.06 (0.12-9.09) | 0.96 | 1 (0.9) | 5 (1.4) | 0.65 (0.08-5.52) | 0.69 |
| Cerebral hemorrhage | 0 | 0 | - | - | 0 | 0 | - | - |
| Myocardial infarction | 1 (1.5) | 4 (1.2) | 1.32 (0.15-11.82) | 0.80 | 1 (0.9) | 4 (1.2) | 0.82 (0.09-7.35) | 0.86 |
| Re-PCI | 2 (2.9) | 11 (3.1) | 0.97 (0.22-4.39) | 0.97 | 1 (0.9) | 11 (3.0) | 0.30 (0.04-2.30) | 0.24 |
| Re-Hospitalization | 1 (1.4) | 8 (2.4) | 0.66 (0.08-5.30) | 0.70 | 1 (0.9) | 9 (2.6) | 0.36 (0.05-2.87) | 0.34 |
| Stent thrombosis | 0 | 2 (0.6) | 0.04 (0-517683.08) | 0.70 | 0 | 2 (0.6) | 0.03 (0-39606.35) | 0.63 |
| CABG | 0 | 0 | - | - | 0 | 0 | - | - |

Data are presented as the number and percentages, along with Kaplan-Meier estimates.

†The confounding factors considered in the IPTW are age, sex, hypertension, diabetes mellitus, current smoking, prior cerebrovascular accident, CHA_2_DS_2_-VASc score, LVEF, HR, peak CK-MB, total cholesterol, LDL-cholesterol, aspirin, clopidogrel and ticagrelor. After matching, all variables were very well balanced.

Abbreviations: OAC, oral anticoagulant; HR, hazard ratio; MACCE, major adverse cardiac and cerebrovascular events; MI, myocardial infarction; TIA, transient ischemic attack; PCI, percutaneous coronary intervention; CABG, coronary artery bypass graft.
